# A novel use of Stereo Baited Remote Underwater Video and Drop-Down Video for biodiversity and marine landscape mapping and prediction

**DOI:** 10.1371/journal.pone.0319355

**Published:** 2025-04-03

**Authors:** Natasha L. Walker-Milne, Sophie Elliott, Peter J. Wright, David M. Bailey

**Affiliations:** 1 School of Biodiversity, One Health and Veterinary Medicine, College of Medical, Veterinary and Life Sciences, University of Glasgow, Glasgow, United Kingdom; 2 Game and Wildlife Trust, Salmon and Trout Research Centre, Wareham, United Kingdom; 3 Marine Scotland Science, Aberdeen, United Kingdom,; 4 Marine Ecology and Conservation Consultancy, Ellon, United Kingdom; King Abdulaziz University, SAUDI ARABIA

## Abstract

To make the best-informed decisions on managing marine resources whilst also restoring biodiversity, the creation and analysis of benthic maps is highly valuable. This study focuses on benthic mapping to study patch - and landscape - level processes affecting epifauna and demersal species using Stereo Baited Remote Underwater Video (SBRUV) and Drop-Down Video (DDV) surveys. Surveys were conducted within the South Arran Marine Protected Area between 2013–2019 and yielded 554 SBRUV data points and 333 DDV data points. These data were processed to determine proportional coverage of substrata and kriged to produce benthic maps. From this it was possible to estimate the patch scale of substrata to gain a more detailed understanding of the marine landscape, composition structure, and epibenthic biodiversity. The resulting validated maps allowed the number of substrata patches and patch characteristics such as area and perimeter-to-area ratio to be calculated to support management and understanding of the value of this seascapes for benthic and demersal biodiversity. Our approach allowed for the identification of areas of higher biodiversity that may assist during mapping of Marine Protected Areas’ (MPA) vulnerable features. These methods should provide better information about site condition and ecosystem service provision than existing methods.

## Introduction

Maps of seabed geology, biogenic habitats and biodiversity are essential to several areas of conservation, including the selection and monitoring of marine protected areas (MPAs), and ecosystem-based fisheries management. Changes in the extent and fragmentation of features such as seagrass or maerl beds are indicators of the success of a protected area designed to conserve these seabed types [1]. To support fisheries, we also need to know where essential fish habitat is and identify measures of the value of different substrata and patch sizes. Environmental conditions, and the patch’s spatial relationship to other patches of the same and different type also play a role, such as how that area is connected to others for the purposes of foraging, migration, and ontogenetic transition [2–4]. Therefore, the idea of a benthic species’ habitat being defined by a single substratum type, as reflected in traditional habitat classification systems like EUNIS (European Nature Information System), develops into a concept where the patchwork of different substrata and environmental conditions must be viewed collectively, the “seascape ecology” approach [5]. This concept is useful, but to “operationalise” seascape ecology we need seabed maps which reveal the mosaic of substrata at scales relevant to the lives of marine animals [6]. Producing maps which encompass sufficient detail may be challenging as, depending on the taxon in question, the necessary resolution could be in metres, with even very narrow barriers/boundaries enormously changing the environment from the perspective of a fish.

Benthic substratum mapping has become essential to informing spatial-based management systems such as MPAs. There are many approaches to conducting underwater surveys for monitoring and mapping the benthic substratum and demersal species, such as trawls, acoustic surveys, drop-down video (DDV), underwater visual census (UVC), baited remote underwater video (BRUV), remote operated vehicles (ROV), and autonomous underwater vehicles (AUV) [7–9]. Each method does have their drawbacks; traditional trawl surveys provide excellent data on species numbers but are destructive to the seafloor, especially delicate ecosystems, and provide no information on seabed composition [10,11]; although acoustic surveys are able to cover large areas, they are limited in their ability to distinguish benthic and demersal species [12]; DDV is very efficient for seabed composition surveys but is limited in its ability to identify fish species [7]. ROVs and AUVs offer access to depths that would be beyond those of diver surveys, but they require specialist equipment and training which can be prohibitive [13]; and diver surveys to conduct UVCs are restricted by practical limits on the depth and duration of dives [7,14]. Baited remote underwater video (BRUV), and their counterpart, Stereo Baited Remote Underwater Video (SBRUV), combine the advantages of DDV whilst being proven to be efficient in both cost and time [15]. However, as with DDV, BRUVs can be impeded by factors such as water turbidity especially in temperate areas when seasonal algal blooms can impact visibility [16], but due to fast simple deployment it is possible to gather additional data relatively easily. SBRUV deployments also fulfil a dual role in capturing both faunal surveys and benthic composition providing additional information on relationships between substrata and species [14,17]. This approach can lead to a combination of seabed predicted mapping and provide additional faunal surveys to produce predictions of demersal and epibenthic species presence - absence and biodiversity [18]. Overall, baited camera setups provide a balanced, cost-effective and non-destructive method for benthic surveying [19].

When examining species presence for calculation of biodiversity, there can be some bias from any surveying method employed. Bait increases the recording of carnivorous/scavenging species without decreasing herbivorous species as other species may become attracted to fish activity regarding the bait [20–22]. SBRUVs when compared to UVC surveys record greater species richness and generalist carnivores [15,23], making them a more useful tool to measure species richness [15]. However, calculations of biodiversity from SBRUVs can be influenced by differences in how species respond to bait [24] and there is evidence that species with increased site fidelity may impact SBRUV sampling [25]. When compared to other survey methods, SBRUVs reduce disturbance when assessed against similar survey approaches such as UVC which can be impacted by diver presence [22,26]. There are also behavioural interactions between species attracted to SBRUV bait with smaller, shyer species reacting negatively to bolder or schooling species [27].

Demersal fish occurrence is influenced by seabed type and its associated biodiversity [18,28]. By increasing shelter and feeding opportunities, heterogeneous marine landscapes can benefit certain species [18,29]. In contrast, species which are highly specialised or adapted to a particular stable substratum may benefit from a more homogenous landscape (e.g., Ewers and Didham [30]). A major threat to benthic biodiversity is substratum fragmentation; the process by which a large area of substratum is broken into smaller, isolated, subunits. Substratum fragmentation can lead to lower fish abundance due to the reduction of seabed quality, biodiversity, shelter and feeding areas [31–33]. Fragmented areas with barriers formed by currents or impassable substratum can provide a selection of habitat subunits where refuges from disturbance can provide a reservoir for repopulation after a disturbance event [34]. However, if the area becomes too fragmented then these subunits may become too small to support a viable population [35]. Anthropogenic actions such as dredging, trawling, anchoring and mineral extraction can lead to losses in some types of seabed substrata [11,36–38]. Certain commercial fishing gear (e.g., scallop dredging and benthic trawls) can impact some habitat-forming taxa such as bivalves, both directly and indirectly even in species for which there is no commercial fishery, such as fan mussels (*Atrina fragilis*) [39]. Furthermore, contact with certain fishing gear can damage other substrata such as seagrass, maerl, and sponges [40–42], leading to a loss of biodiversity [43–45]. Therefore, conserving the remaining seabed and the issue of fragmentation, are inexorably linked as species richness is negatively affected by reductions in both substratum quality and increased fragmentation [46–48]. Effective monitoring and mitigation of these impacts require precise mapping of seabed types, which can identify areas most at risk from fragmentation. Advanced spatial techniques such as kriging play a crucial role as they enable the prediction of habitat continuity and substratum coverage even in areas with limited direct survey data. By providing detailed, interpolated maps, kriging helps to reveal patterns of habitat fragmentations and connectivity, guiding conservation strategies and informing management decisions. Such predictive approaches are essential for conservation planning ensuring that interventions are targeted effectively [6,33].

**Fig 1:**
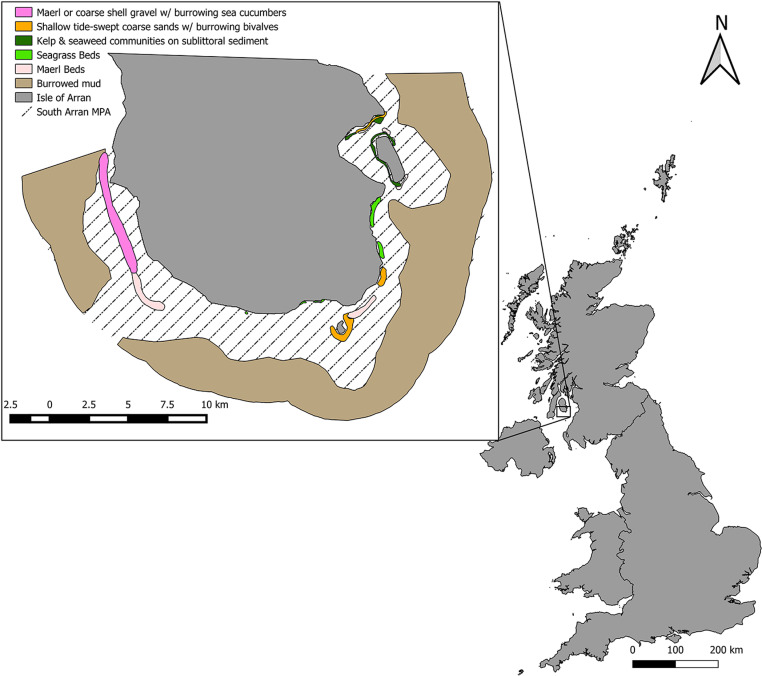
The South Arran Nature Conservation MPA features when designated. Colours indicate the known substrata prior to designation, most of which are designated “features” intended to be conserved or restored. Zoomed out map shows the position of South Arran relative to the UK [52].

We used a combination of SBRUV and DDV imaging techniques to map seabed landscape, including substratum type, patch shape, and form. Our approach was applied to the South Arran MPA as a case study to highlight its potential uses in coastal marine management. The South Arran Nature Conservation MPA was designated in 2014 by the Scottish government and management measures came into force in 2016 [49]. The South Arran MPA contains eight protected features which are listed for either conservation or recovery (Fig 1). Within South Arran MPA, there are a number of different management measures which limit various types of fishing activity including the Lamlash Bay No Take Zone (NTZ) that was established in 2008 and encompasses 2.67 km^2^ and is the only fully no take area designated in Scotland [50]. Previously, seabed analysis of the MPA area was conducted using DDV and diver surveys [51], our methods improve on previous data by recording species at the same time as seabed composition. Subsequent maps were then used to compare demersal and epibenthic biodiversity (hereafter referred to collectively as the biodiversity) between different management zones within the South Arran MPA, illustrating a practical application of surveying using readily available equipment for informing management decisions. As such our study aims are as follows: 1) Make and validate maps of substrata; 2) Extract landscape metrics from maps; 3) Map biodiversity within the MPA, 4) Compare the biodiversity in MPA zones with different fishing restrictions.

## Materials and methods

### Study area and surveying

**Fig 2:**
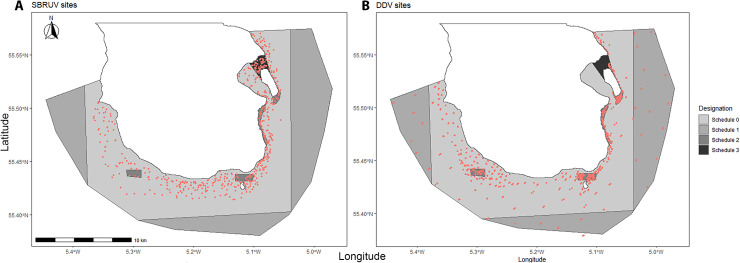
Locations of all Stereo baited remote underwater video and drop-down video (DDV) surveys. **(A)** University of Glasgow SBRUV survey sites for all years 2013-2019. **(B)** NatureScot drop-down video (DDV) survey locations for 2013-2018. fishing management limitations are as follows outlined in Table 1.

The seabed within the South Arran MPA consists of a mixture of sediments ranging from burrowed mud to sand with seagrasses and had previously been surveyed by NatureScot with additional dive surveys conducted by the Community Of Arran Seabed Trust (COAST) and Seasearch data in order to identify MPA “search features” before the designation of the MPA. This map was then used to support decisions on management zones within the MPA (Figs 1 and 2) [51].

**Table 1:** Fishing restrictions implemented in the South Arran MPA as per marine conservation order 2015. TND) Trawl and Dredge - Limitations throughout MPA: no demersal trawling and scallop dredging. BT) Beam Trawl - As with TND but with restrictions on beam trawling and limited demersal trawling allowed. DTDC) Demersal Trawl Dredge and Creel - As with TND but with additional bans on passive gear such as creels. NTZ) No fishing activities whatsoever – (No Take Zone).

Schedule (ordered least-most restrictive)	Gear prohibited				
	Scallop dredge	Demersal trawl	Otter trawl, “specified vessels”	Passive gear (e.g., creeling)	Scallop diving
1 (trawl derogation - BT)	x	x			
0 (core - TND)	x	x	x		
2 (maerl boxes - DTDC)	x	x	x	x	
3 (NTZ)	x	x	x	x	x

Fishing activities of various types are permitted within the South Arran MPA, but since the MPAs designation, fishing management restrictions have been implemented that limit certain activities. These fisheries management measures comprise a core area of the MPA where trawling, dredging, and otter trawls are prohibited (TND); an outer region further from shore where dredging and trawling are prohibited (BT); several areas where only passive gear is permitted (DTDC); and a full no take zone where all activities including scallop diving and prohibited (NTZ) (Table 1).

**Fig 3:**
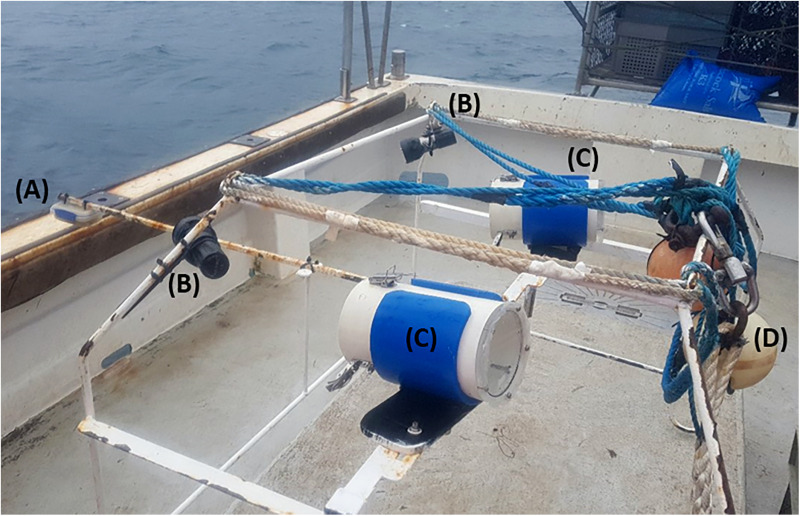
Stereo baited remote underwater video setup ready for deployment. **(A)** bait box **(B)** W38VR Archon lights **(C)** underwater housings containing Canon HF G25 video cameras **(D)** float and ropes to deploy frame.

SBRUV surveys were carried out within the South Arran Marine Protected Area in 2013, 2014, 2018 and 2019, using a 6.5 m RIB, 10.8 m research vessel (RV Actinia), and 10.0 m creel boat (Table 2). The study area was divided into 5 zones differing in exposure level and topography [4], stations were generated within each zone via random points stratified by depth using Quantum Geographic Information System [53], and identified using combinations of alphanumeric identifiers including year and date. The three SBRUV camera set ups each consisted of a pair of Canon HF G25 video cameras encased in waterproof camera housings with an inward angle of ~ 8^o^ and a basal separation of 58 cm [4,54], mounted on a steel frame 57 cm in height, with two LED W38VR Archon lights providing illumination (Fig 3). All cameras were set to automatic exposure, with focus set to infinity. Frames were freshly baited with locally caught mackerel (*Scomber scombrus*) for each station [4]. The camera systems remained on the seabed and recording for 60 minutes per deployment.

**Table 2:** Survey details from 2013–2019. All Stereo Baited Underwater Video (SBRUV) and drop-down video (DDV) surveys carried out between 2013–2019, including vessels used, camera equipment, depth, and times.

Survey method	Year	Vessel	Camera Equipment	Min Depth (m)	Max Depth (m)	Earliest Start Time (HH:MM)	Latest Start Time (HH:MM)
*SBRUV*	2013	6.5 m RIB	Canon HF G25	5	29.1	08:01	14:29
	2014	RV Actinia 10.8 m	Canon HF G25	4.1	47.2	07:52	14:44
	2018	10.0 m creel boat	Canon HF G25	5.82	45.9	08:47	16:25
	2019	10.0 m creel boat	Canon HF G25	5.49	40.7	08:51	16:27
*DDV*	2013	MRV Sir John Murray	Kongsberg OE14–366	9.3	107.4	08:20	15:58
	2014	RV Kelpie 7.9 m	Outland SD camera	6	31	09:41	17:18
	2018	Non-specialised fishing vessel	C-Tecnics ST 3023	2	27	08:21	16:46

In order, to prevent bait odour plumes from affecting neighbouring deployments on the same day camera systems were positioned more than 500 metres apart, depths for SBRUV deployments ranged from 4.1 m to 47.2 m. Our sampling for diversity may have been affected by the time of day of deployments, which were all conducted between 07:45 and 17:28 (all daylight hours), thus excluding any nocturnal sampling, as times for deployment were limited to boat availability and tides.

Drop-Down Video (DDV) surveys were commissioned by Marine Scotland (now the Marine Directorate) with NatureScot, surveys were carried out in March and July 2013 aboard the Scottish Environmental Protection Agency (SEPA) research vessel MRV Sir John Murray (Table 2). In September of 2014 surveys were carried out onboard SEPA survey vessel [55]. Surveys in September 2018 were carried out using a fishing vessel [56] (Table 2). DDV cameras were used in accordance with recommending operating procedures as per JNCC guidelines [57,58], with camera high above the seabed maintained using laser measurements for consistency.

**Fig 4:**
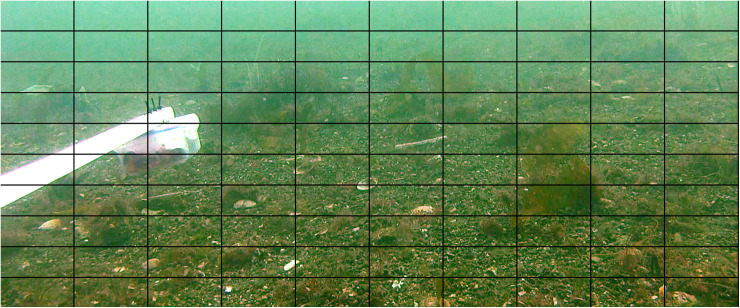
Grid overlay (10 × 10) of stereo baited remote underwater video (SBRUV) image seen from right hand camera. Screen grab of right hand SBRUV camera showing 10 × 10 grid overlay of image depicting substrata composition of South Arran survey point SA_201, each grid cell was classified by the substrata which comprised >  50% of each grid cell. SBRUV bait arm is present on the left of the image.

**Fig 5:**
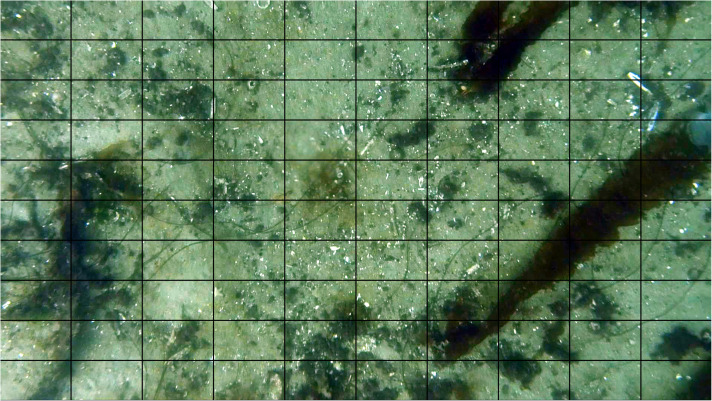
Grid overlay (10 × 10) of drop-down video (DDV). Screen grab from DDV camera showing 10 × 10 grid overlay of image depicting substrata composition of South Arran survey point Station 11a, each grid cell was classified by the substrata which comprised >  50% of each grid cell.

Examination of the substratum was carried out for each SBRUV and DDV deployment, SBRUV frames were angled at ~ 15^o^ oblique view to achieve a partial angle of the seabed as per [4] (Fig 3); DDV images differed from the SBRUV images due to the line of sight being perpendicular to the seafloor. Videos were analysed using Event Measure™ software [54] with a 3 minute lead in time allowing for sediment to settle. Prior to deployment, all SBRUV cameras systems were calibrated, measurements were conducted within confined controlled water and calibrated using CAL software (version 3.30) [59]. The maximum number of individuals present on the screen at any one time (MaxN) was recorded for each species. MaxN was determined by reviewing the footage and identifying the single frame in which the highest number of individuals of a given species were simultaneously visible, in order to ensure consistency and avoid overestimation. A still image was captured from each station for substratum analysis at the beginning of each deployment. Each still image from SBRUV was cropped to a set aspect ratio that included the best average coverage, calculated by taking a random selection of 50 screengrabs and calculating the average aspect ratio that would include the aspect ratio that encompassed the best field of view (FOV) to capture both seabed and fauna, the resulting images was overlaid with a grid of 10 x 10 cells (DDV images being perpendicular to the seafloor did not require cropping) (Figs 4 and 5). The substratum that comprised the majority within each grid square was recorded to create a proportional coverage of each substratum type at each station. Grid squares that could not be identified due to poor image clarity or obstruction were classified as unknown and excluded. Substrata were classed as algae, seagrass, maerl, gravel, mud, sand, boulder, and pebble with size based on measurements taken using EventMeasure™ [54]. Some substratum types such as boulders, seagrass, maerl, both living and dead, were too sparse in their occurrence to successfully produce robust data sets and were combined into a single category (e.g., maerl into the ‘gravel’ substratum type (Table 3)). Dead maerl, however, may also play an important role in the provision of interstitial spaces for a variety of species. Species of red, brown, and green algae were merged into a single designation to account for all algal types encountered. Still images were captured from all DDV deployments, two still images were used from each deployment, one at the drop and one from the haul, as these points could be geolocated using the latitude and longitude for the start and end of the tow. Each image was then split into 100 grid cells, as per the SBRUV images, and the proportional coverage of each image was calculated using the coverage of substrate in each cell. Species were not recorded using DDV as the movement across the seafloor for recording substratum is continuous, and DDV has a smaller field of view, DDV were only used for substratum analysis.

**Table 3:** Sediment size and classification †maerl could not be taken into consideration independently due to the small area.

Sediment Type	Sediment composition	Expected Particle size (cm)
Pebble	Small to medium stones and shells	~4–6
Gravel	Mixture of stone, shell and maerl †	~2 - 4
Sand	Soft sedimentary sand including seagrass when present	~ 0.1–2
Mud	Silty sediment high period of water column suspension	<0.1

### Biodiversity and seabed mapping

Using the maximum number of individuals of a species captured in each SBRUV deployment (MaxN), both mobile demersal (e.g., *Gadus morhua, Eutrigla gurnardus, Limanda limanda*) and epibenthic species (e.g., *Asterias rubens, Ophiothrix fragilis, Aphrodita aculeata*) were identified to species level (Table S1), in order to calculate biodiversity for each site, all modelling was carried out using R software [60], and Simpson’s diversity was calculated for each SBRUV deployment using the rdiversity package [61]. Simpson’s diversity Index was used as a measure for biodiversity as it provides average proportional abundance of each species within a dataset. Biodiversity was then interpolated using ordinary kriging across the whole extent of the MPA. Diversity and substratum coverage for each site was predicted using ordinary kriging, including standard error predictions (Fig S1), in the R package automap [62] to estimate proportional substratum coverage throughout the area of the MPA. Maps and kriged calculations were conducted using a combination of the rgdal [63], rgeos [64], gstat [65], geosphere [66], and raster [67] packages. As data was irregular the package akima [68] was used to create rasters for mapping, and figures were produced using the ggplot2 package [69]. Variograms were produced and 5-fold cross validation undertaken to examine Mean Error (ME), Mean Predicted Standard Error (MPSE), Mean Square Normalised Error (MSNE), Correlation Observed and Predicted (COP), and Correlation and Predicted Observed (CPR) (Table S2). Differences in biodiversity between substratum classifications and fishing management zones within the MPA were compared using a pairwise comparison, as the data did not meet the normality and homogeneity of variance assumptions for an ANOVA test non-parametric tests were conducted including Kruskal-Wallis, Wilcox and post-hoc Dunns tests using the rstatix [70], and the multcompView [71] package for visualisation.

### Marine landscape analysis

From the kriged predictions of proportional coverage for each substratum type, each substratum type was aggregated into distinct patches to examine patch characteristics such as patch area, ratio of perimeter to area (RPA) and fractal dimension index using SDMTools R package [72]. Unlike remote sensed multiband layers which can only have a 0 or 1 value for presence/absence of substratum type, the results in this study have cells which could theoretically be made up of a mix of all substratum types. Therefore, the base level threshold for mapping was set to 0.2 proportional coverage as five substratum classifications were used. This would also reduce inflating patch size where sporadic substratum occurrences were found. Patch overlap may occur where survey sites revealed locations that had a mix of substratum types. Patch analysis was conducted using a 500 m x 500 m grid overlay on the predicted substrata maps and calculating a mean value contained within each deployment (Fig S2). Fractal dimension index (FDI) ranged from 0–2 and is a unitless measure of edge complexity calculated from the base 2 log of the proportional increase in information obtained when the edge of the patch is evaluated at increasing spatial resolutions, a greater value indicates more complex edge characteristics [73, 74].

## Results

In total 511 SBRUV deployments were conducted between June and September from 2013 to 2019 (Table S3). 57 species were identified across all sites; these included a range of demersal and epibenthic species (Table S1)*.* The DDV surveys resulted in 333 sites carried out between 2013 and 2018.

### Biodiversity and seabed mapping

**Fig 6:**
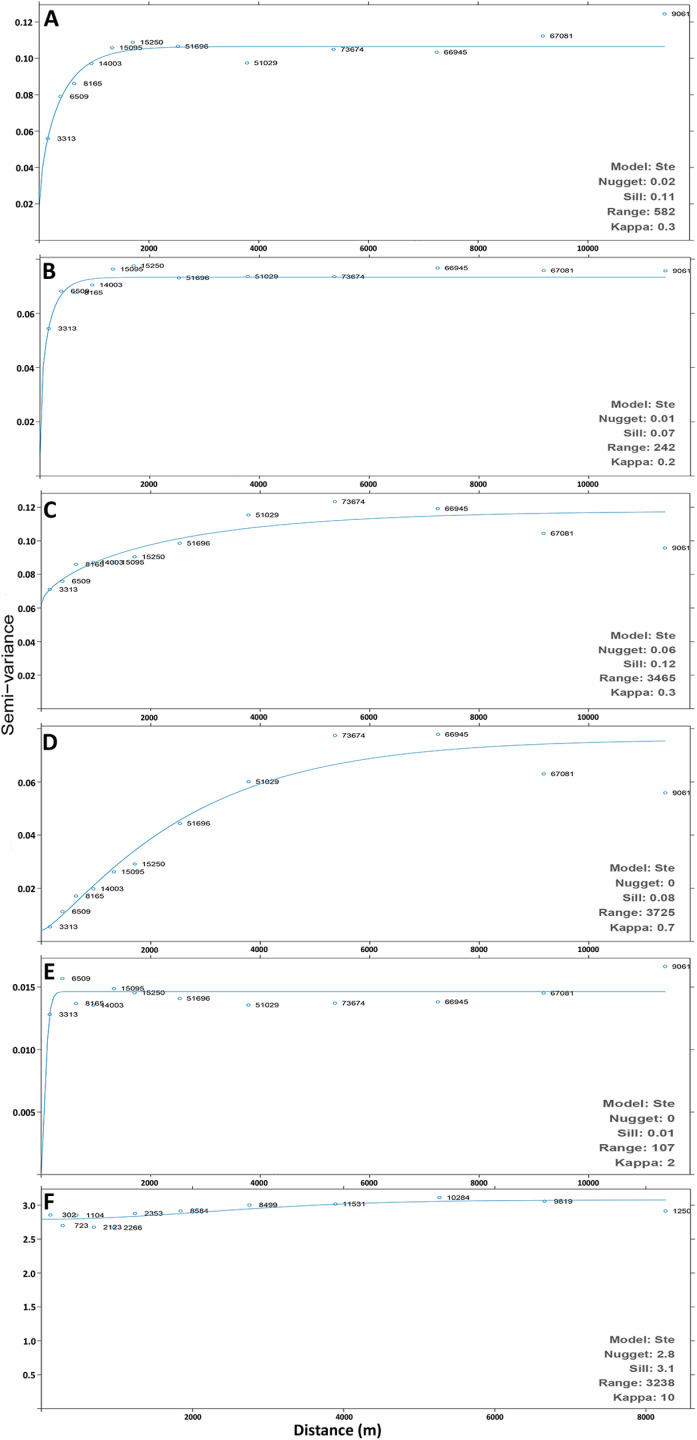
Directional variogram for the predicted proportional coverage of substratum. Variograms produced from ordinary kriging of each substratum: **(A)** Algae **(B)** Gravel **(C)** Sand **(D)** Mud **(E)** Pebble in metres (m) and **F)** Inverse Simpson’s Diversity Index.

Kriged prediction including standard error predictions were produced (Fig S1), and to examine the confidence of our predictions relating to spatial variance between data points, variograms of the kriged predicted maps were also produced (Fig 6, Table S2). Variogram results indicate different spatial characteristics across substratum types. Algae exhibited a short range before reaching the variogram sill, suggesting limited spatial continuity in its distribution. Sand and mud showed a larger range, reflecting a more widespread and continuous spatial pattern. Biodiversity displayed the highest nugget effect, indicating substantial small-scale variability within the sampling points (Fig 6).

**Fig 7:**
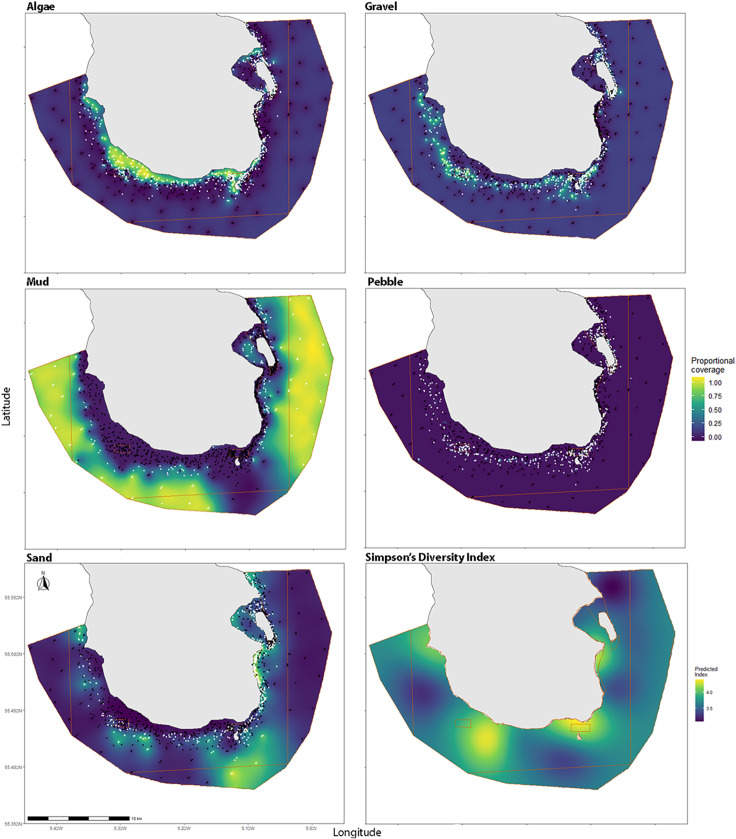
Maps of predicted substratum coverage produced from ordinary kriging. Maps were produced using ordinary kriging from data from stereo baited remote underwater video and drop-down video surveys. White points denote presence of substratum type, black is an absence of substratum type, orange bounding boxes outline MPA fishing restrictions (as described in Table 1).

Predicted proportional coverage of algae ranged from 0–0.95, the largest extent of algae stretched around the southwest coast of the island (Fig 7). Gravel (which also contained some maerl both living and dead), ranged from 0–0.76 proportional coverage and was mainly located in deeper areas adjacent to algae stretching along the southern and western coasts. Areas of sand were located to the east of the island (which also contained some seagrass) and to the south, as well as several small patches throughout the MPA.

**Fig 8:**
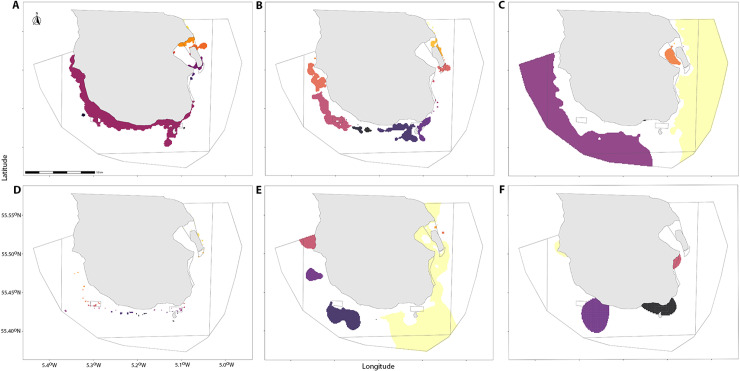
Predicted substrata patches within the South Arran MPA. Patches of predicted substrata **(A)** Algae, **(B)** gravel, **(C)** mud, **(D)** pebble, **(E)** sand and **(F)** highest Inverse Simpson’s Diversity (top 20% of Inverse Simpson’s Diversity Index, areas with value greater than threshold of 4.04). Landscape patches are coloured individually to illustrate separation, bounding boxes outline MPA fishing restrictions (as described in Table 1).

Substrata predictions which met the 0.2 threshold of coverage were divided into patches. Pebble had the most fragmented composition with a total of 49 patches, whereas mud was the most continuous with the majority of coverage contained within two large areas extending beyond the boundary of the MPA (Fig 8).

### Marine landscape analysis

**Fig 9:**
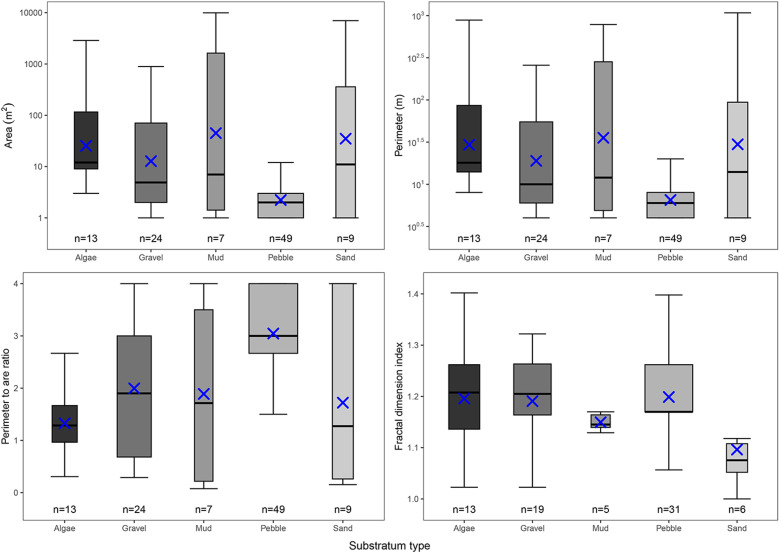
Patch characteristics per substratum Characteristics of substrata patches for area (m^2^), Perimeter **(m)**, Perimeter to area ratio, and fractal dimension index. Blue crosses denote the median and bars indicted the mean, boxplot width denotes number of data points.

Mud patches combined had the largest overall area (18935 m^2^ in total across seven patches) and pebble the lowest (147 m^2^ across 49 patches), but pebble had the highest perimeter to area ratio (RPA) (3.05 SE ± 0.117) followed by gravel (2.00 SE ± 0.270) (Fig 9). Sand had the largest range in perimeter to area ratio (minimum RPA 0.154, maximum RPA 4 – Interquartile range (IQR) 3.74) showing a high degree of variability across only nine distinct patches.

**Fig 10:**
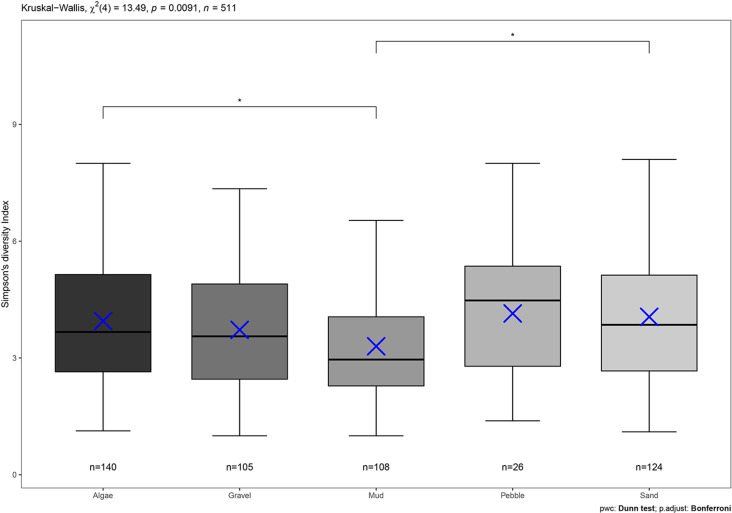
Inverse Simpson’s diversity index for each substratum and pairwise comparison. Inverse Simpson’s diversity index of each substratum. Pair wise comparisons made between substrata regarding biodiversity indicate statistical significance difference between algae to mud and sand to mud. Each datapoint is one SBRUV station, categorised by the most common substratum type observed. Blue crosses denote the median and bars indicated the mean, boxplot width denotes number of data points.

Four areas of increased biodiversity were identified, these were areas of the upper twenty percent of predicted Inverse Simpson diversity (mean =  3.69, upper 20% >  4.04). Mud and sand had the largest patch areas with a maximum proportional coverage of 1.0 and 0.77 respectively. When examining differences in demersal and epibenthic biodiversity between substrata patches the results of the Kruskal-Wallis chi-squared test were significant (H =  0.018, df =  4, *P* =  0.009), and a pairwise post-hoc Dunn’s test with a Bonferroni adjustment showed that biodiversity was statistically significant between the different substratum types (Mud – Algae, z =  -2.9, *P* =  0.03; Mud – Sand, z =  3.24, *P* =  0.001) (Fig 10).

**Fig 11:**
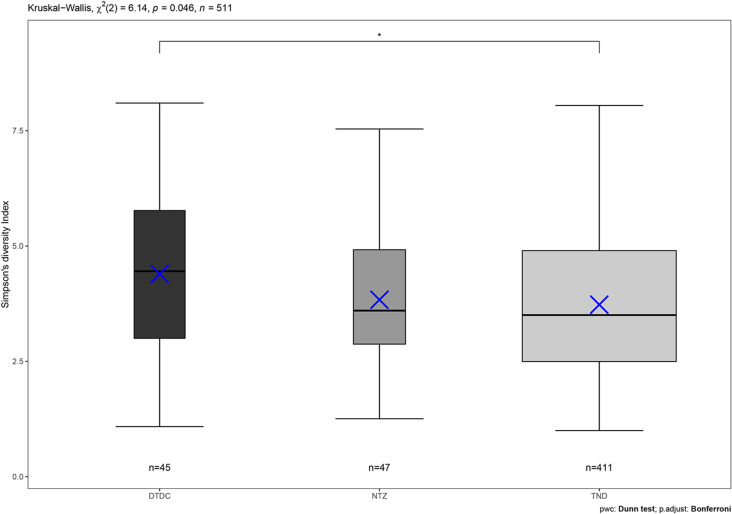
Inverse Simpson’s diversity index for each fishing limitation area. Inverse Simpson’s diversity index for each type of fishing limitation within the MPA study area. Pair wise comparisons made between substrata regarding biodiversity indicate statistical significance difference between areas closed to dredging, trawling and creeling (DTDC), and areas closed to trawling and dredging (TND). Each datapoint is one SBRUV station. Blue crosses denote the median and bars indicted the mean, boxplot width denotes number of data points.

When diversity is compared between fishing measures limitations areas within the South Arran MPA, there is a statistically significant difference between the core area of the MPA which has restrictions on trawling and dredging (TND), and the areas closed to trawling, dredging and creeling (DTDC). Kruskal-Wallis chi-squared test were significant (H =  0.008, df =  2, *P* =  0.046), and a pairwise post-hoc Dunn’s test with a Bonferroni adjustment (DTDC – TND, z =  -2.42, *P* =  0.04) (Fig 11). As no SBRUV drops were conducted in the bottom trawl derogation (BT) areas there is no comparison between BT and other areas.

**Fig 12:**
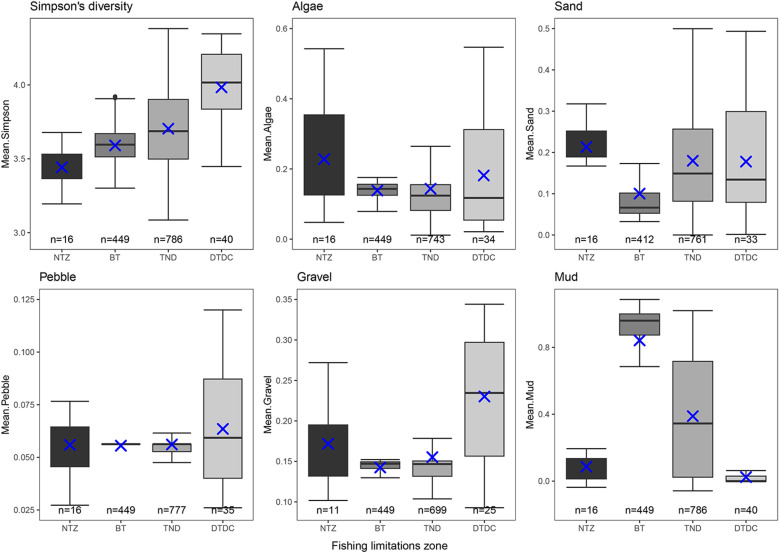
Results from 500 x 500 m gridded breakdown of predicted substrata and diversity within the MPA study area. Mean proportional coverage of each substratum type and mean Inverse Simpson’s Diversity Index for each gridded 500 m x 500 m square (Fig S2) in the South Arran MPA broken down by fishing management limitations (Fig 2, Table 1). Blue crosses denote the median and bars indicted the mean.

Predicted proportional coverage of each substratum and biodiversity throughout the MPA calculated by mean per 500 x 500m grid square (Fig S2) is shown in Fig 12. Areas closed to all bottom trawling but still open to static gear such as creeling (TND, Fig 2, Table 1), had the highest mean biodiversity. Areas closed only to demersal and beam trawling had lower biodiversity, but as these covered larger areas had move overall variation (Fig 12). The area closed to all fishing activities had the lowest biodiversity, but as this only encompasses 2.67 km^2^ it will not possess as much variation in substrata and overall surface area. Having only been closed to all fishing activities in 2008 it will be interesting to see how this develops in the future.

**Fig 13:**
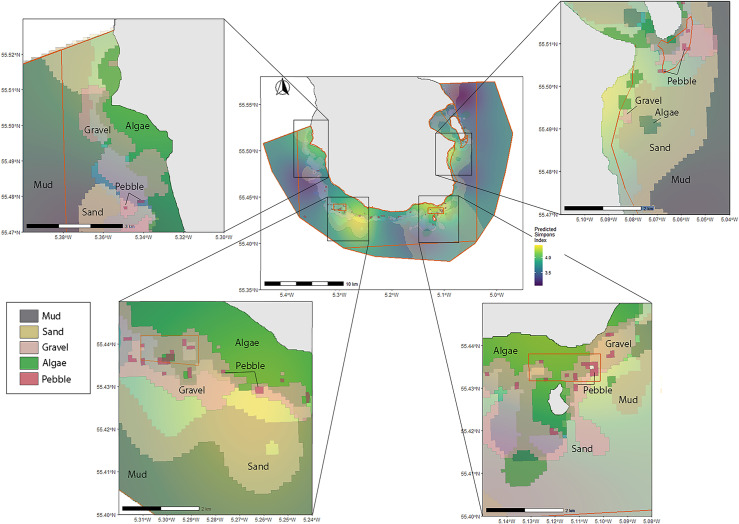
Predicted seabed throughout the MPA with four expanded areas showing higher predicted biodiversity. Map of all substratum types overlayed with predicted Inverse Simpson’s diversity index, showing four zooned in areas with higher biodiversity. Orange boundaries denote areas of differing fishing restrictions as outlined in Fig 2. Areas of higher predicted biodiversity zoomed in to illustrate composition of substratum.

The four areas with the highest predicted biodiversity are in areas where there is a high mix of substratum types, with lower diversity found in areas of extended mud (Fig 13). All patches of substratum are shown to be more fragmented than originally predicted in 2013 apart from burrowed mud which is comparable.

## Discussion

In order to identify and monitor the factors which contribute to improving the quality of the seabed and its diversity, high resolution benthic substratum layers are needed. This study shows that comprehensive fine-scale mapping of demersal and epibenthic composition relevant to landscape scale monitoring is possible using a combination of video techniques. These subsequent maps can then be used to extract valuable landscape metrics, examine and map biodiversity, and compare biodiversity between different fishing management areas. [48,75]. Resolving the extent, and fragmentation of features also provides a means to assess whether Marine Protected Areas conserve or restoration objectives have been achieved. With increasing interest in the marine biodiversity credit sector offering a possible source of conservation funding [76], accurate mapping of benthic areas with benthic and epibenthic biodiversity is of importance. The success or failure of the any such undertaking will hinge on the design being supported by rigorous scientific data for the size and location of conservation credit area placement, the enforcement of management, and adequate resources [77,78]. The method described in this study has the potential to provide valuable data for the informing and development required of any such design and implication. Previous studies have employed SBRUV systems to assess species richness across various marine ecosystems, focusing primarily on habitat associations defined by a predominant substratum type [15,18]. By employing this more comprehensive approach, the study not only enhances our understanding of species-habitat relationships but also allows for a more precise comparison with existing biodiversity and habitat maps.

**Fig 14:**
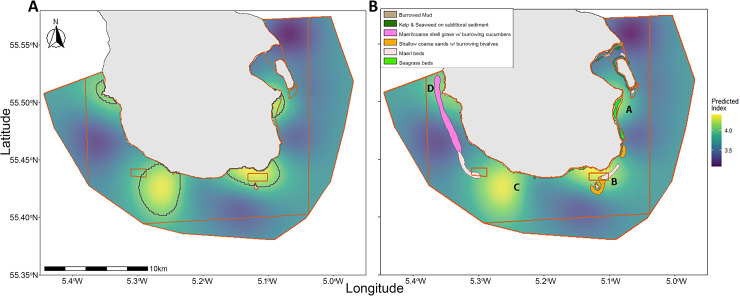
Comparison of predicted Inverse Simpson’s diversity index and original features from MPA designation. **(A)** Predicted biodiversity with increased biodiversity patches outlined (top 20% of Inverse Simpson’s Diversity Index, areas with value greater than threshold of 4.04). **(B)** Overlay of substrata features from NatureScot 2013 survey [52], showing extent of proposed features as of MPA establishment (Fig 1), with predicted Inverse Simpson’s Diversity Index. Four areas of predicted higher biodiversity are denoted as areas A-D. Orange boundaries denote areas of differing fishing restrictions as outlined in Table 2.

When the predicted areas of increased biodiversity are compared to the original predicted substratum features from NatureScot 2013 survey, there is an overlap in identified marine features such as seagrass that coincides with an area of increased biodiversity (Fig 14). There is also an area of increased biodiversity that does not overlap with a protected feature. Predictions of the size and extent of the gravel beds (which also contained maerl both alive and dead), approximately corresponded with the map of the maerl or coarse gravel with burrowing sea cucumbers from the original proposition [51]. However, our results study found several distinct patches rather than a continuous extent of maerl gravel. Our results also correspond with predictions from another study in the Clyde examining distribution of benthic sediment, with our predictions of sand, mud and gravel aligning with their results [79]. This new information will provide a more sensitive measure of change in this feature over time. The degree of impact that marine landscape composition has on species assemblages will depend on the species [80]. Although there may be increases in biodiversity in edge areas [81], if a high degree of fragmentation leads to patches which are too small, predation can have a negative impact on some demersal species [82,83].

Our results show that the highest predicted biodiversity was associated with a high mix of substratum types, with lower diversity found in areas of extended mud, as found in some other studies [33]. The complex structures formed by maerl provide refuge for many species including juvenile fish [84–86]. Valuable interstitial structures, such as those provided by maerl, are sensitive due to their vulnerability to degradation and fragmentation through practices such as trawling and dredging [40,87,88]. Other substrata that were found in close association with areas of maerl gravel were algal dominated areas (Fig 13). There may be a combination of algal/maerl gravel/sand association that provides the best opportunities for the highest number of taxa in foraging opportunities, shelter, area, and composition [89–91]. When examining differences in biodiversity between substrata, algae and sand displayed statistically significant differences to mud. This aligns with previous work illustrating how seabed composition may drive demersal and epibenthic species present [18].

Kriged predictions of biodiversity, as diversity was measured from SBRUV deployments alone, resulted in all data being gathered solely from sites closer inshore and as such predictions into deeper water further from shore are made with increasing standard error (Fig S1). Although the techniques outlined in this study does give an estimation of potential changes in biodiversity, a modelling approach of biodiversity prediction would be more accurate. However, this study does provide robust predicted substrata maps as variables to feed into such models. The characteristics of substrata patches produced in this study could also be examined in conjunction with connectivity to ascertain which combination of patches, size, complexity, and their relationship to each other influences biodiversity.

This study demonstrated that it is possible to broadly identify spatial variation in biodiversity at a landscape scale and examine patch characteristics as a potential measure of seabed fragmentation. Detailed substratum data offers a benchmark for monitoring future substratum changes which can lead to degradation and substratum loss. Although outside the scope of this present study, future work may be carried out to investigate the seabed composition (regarding seabed type, patch characteristics, and connectivity) which improves epibenthic and demersal biodiversity. The advantage of this method of surveying is that relatively simple inexpensive equipment together with data processing and analysis, can be used to create detailed maps of substratum composition. This study provides a valuable tool for easy monitoring of critical substrata for conservation and restoration purposes which is especially beneficial given the poor state of benthic epifauna linked to fishing impacts [92] in the current climate where seabed integrity is under pressure.

## Supporting Information

Table S1 Species list with conservation status, Scottish Marine Priority Marine Feature (PMF) and OSPAR location where species is in decline.
(XSLX)

Table S2 Results of ordinary kriging cross validation for each predicted substrata variable including Mean Error (ME), Mean Predicted Standard Error (MPSE), Mean Square Normalised Error (MSNE), Correlation Observed and Predicted (COP), and Correlation and Predicted Observed (CPR).
(XSLX)

Table S3 Camera surveying deployments carried out between 2013–2019, undertaken by University of Glasgow, Marine Scotland and NatureScot.
(XSLX)

Fig S1 Predictions and standard error maps of substrata. Kriged predictions of substrata coverage and corresponding kriging standard errors maps.
(TIF)

Fig S2 Map of South Arran MPA with 500 m x 500 m gridded overlay. Gridded overlay of the South Arran MPA with the predicted Inverse Simpsons Diversity (Fig 7). Orange bounding boxes outline MPA fishing restrictions (as described in Table 1)
(TIF)

## References

[1] Hennicke J, Blanchard S, Chaniotis P, Cornick L, Hauswirth M, Schellekens T, et al. Report and assessment of the status of the OSPAR network of Marine Protected Areas in 2021. London: OSPAR Commision; 2022.

[2] Cote D, Moulton S, Frampton PCB, Scruton DA, McKinley RS. Habitat use and early winter movements by juvenile Atlantic cod in a coastal area of Newfoundland. J Fish Biol. 2004;64(3):665–79. doi: 10.1111/j.1095-8649.2004.00331.x

[3] Persson A, Ljungberg P, Andersson M, Götzman E, Nilsson P. Foraging performance of juvenile Atlantic cod Gadus morhua and profitability of coastal habitats. Mar Ecol Prog Ser. 2012;456:245–53. doi: 10.3354/meps09705

[4] Elliott S, Turrell W, Heath M, Bailey D. Juvenile gadoid habitat and ontogenetic shift observations using stereo-video baited cameras. Mar Ecol Prog Ser. 2017;568:123–35. doi: 10.3354/meps12068

[5] Nagelkerken I, Sheaves M, Baker R, Connolly RM. The seascape nursery: a novel spatial approach to identify and manage nurseries for coastal marine fauna. Fish and Fisheries. 2013;16(2):362–71. doi: 10.1111/faf.12057

[6] Swanborn DJB, Huvenne VAI, Pittman SJ, Woodall LC. Bringing seascape ecology to the deep seabed: A review and framework for its application. Limnol Oceanograp. 2021;67(1):66–88. doi: 10.1002/lno.11976

[7] Murphy HM, Jenkins GP. Observational methods used in marine spatial monitoring of fishes and associated habitats: a review. Mar Freshwater Res. 2010;61(2):236. doi: 10.1071/mf09068

[8] Bongiorno DL, Bryson M, Bridge TCL, Dansereau DG, Williams SB. Coregistered hyperspectral and stereo image seafloor mapping from an autonomous underwater vehicle. J Field Robotics. 2017;35(3):312–29. doi: 10.1002/rob.21713

[9] Micallef A, Le Bas TP, Huvenne VAI, Blondel P, Hühnerbach V, Deidun A. A multi-method approach for benthic habitat mapping of shallow coastal areas with high-resolution multibeam data. Continental Shelf Research. 2012;39–40:14–26. doi: 10.1016/j.csr.2012.03.008

[10] Hall-Spencer J, Allain V, Fosså JH. Trawling damage to Northeast Atlantic ancient coral reefs. Proc Biol Sci. 2002;269(1490):507–11. doi: 10.1098/rspb.2001.1910 11886643 PMC1690925

[11] Auster PJ, Malatesta RJ, Langton RW, Watting L, Valentine PC, Donaldson CLS, et al. The impacts of mobile fishing gear on seafloor habitats in the gulf of Maine (Northwest Atlantic): Implications for conservation of fish populations. Reviews in Fisheries Science. 1996;4(2):185–202. doi: 10.1080/10641269609388584

[12] Horne JK. Acoustic approaches to remote species identification: a review. Fisheries Oceanography. 2000;9(4):356–71. doi: 10.1046/j.1365-2419.2000.00143.x

[13] Barrett N, Seiler J, Anderson T, Williams S, Nichol S, Hill S. Autonomous underwater vehicle (AUV) for mapping marine biodiversity in coastal and shelf waters: Implications for marine management. Sydney: OCEANS’10 IEEE; 2010. p. 1–6.

[14] Cappo M, Harvey E, Malcolm H, Speare P. Potential of Video Techniques to Monitor Diversity, Abundance and Size of Fish in Studies of Marine Protected Areas. In: Beumer JP, Grant A, Smith DC, editors. Aquatic protected areas What works best and how do we know? Queensland: University of Queensland; 2003. p. 455-64.

[15] Langlois T, Harvey E, Fitzpatrick B, Meeuwig J, Shedrawi G, Watson D. Cost-efficient sampling of fish assemblages: comparison of baited video stations and diver video transects. Aquatic Biology. 2010;9(2):155–68. doi: 10.3354/ab00235

[16] Bicknell A, Godley B, Sheehan E, Votier S, Witt M. Camera technology for monitoring marine biodiversity and human impact. Frontiers in Ecology and the Environment. 2016;14(8):424–32. doi: 10.1002/fee.1322

[17] Harvey E, Newman S, McLean D, Cappo M, Meeuwig J, Skepper C. Comparison of the relative efficiencies of stereo-BRUVs and traps for sampling tropical continental shelf demersal fishes. Fisheries Research. 2012;125:108–20. doi: 10.1016/j.fishres.2012.01.026

[18] Elliott SAM, Sabatino AD, Heath MR, Turrell WR, Bailey DM. Landscape effects on demersal fish revealed by field observations and predictive seabed modelling. PLoS One. 2017;12(12):e0189011. doi: 10.1371/journal.pone.0189011 29228035 PMC5724865

[19] Bailey D, King N, Priede I. Cameras and carcasses: historical and current methods for using artificial food falls to study deep-water animals. Marine Ecology Progress Series. 2007;350:179–91. doi: 10.3354/meps07187

[20] Cappo M, Harvey E, Shortis M, editors. Counting and measuring fish with baited video techniques-an overview. Australian Society for Fish Biology Workshop Proceedings. Tasmania: Australian Society for Fish Biology; 2006.

[21] Harvey ES, Cappo M, Butler JJ, Hall N, Kendrick GA. Bait attraction affects the performance of remote underwater video stations in assessment of demersal fish community structure. Marine Ecology Progress Series. 2007;350:245–54. doi: 10.3354/meps07192

[22] Watson D, Harvey E, Anderson M, Kendrick G. A comparison of temperate reef fish assemblages recorded by three underwater stereo-video techniques. Marine Biology. 2005;148(2):415–25. doi: 10.1007/s00227-005-0090-6

[23] Cappo M, Speare P, De’ath G. Comparison of baited remote underwater video stations (BRUVS) and prawn (shrimp) trawls for assessments of fish biodiversity in inter-reefal areas of the great barrier reef marine park. Journal of Experimental Marine Biology and Ecology. 2004;302(2):123–52. doi: 10.1016/j.jembe.2003.10.006

[24] Unsworth RKF, Peters JR, McCloskey RM, Hinder SL. Optimising stereo baited underwater video for sampling fish and invertebrates in temperate coastal habitats. Estuarine, Coastal and Shelf Science. 2014;150:281–7. doi: 10.1016/j.ecss.2014.03.020

[25] Ghazilou A, Shokri M, Gladstone W. Comparison of baited remote underwater video (BRUV) and underwater visual census (UVC) for assessment of reef fish in a marginal reef in the northern Persian Gulf. Iranian Journal of Ichthyology. 2019;6(3):197–207. doi: 10.22034/iji.v6i3.353

[26] Lowry M, Folpp H, Gregson M, Suthers I. Comparison of baited remote underwater video (BRUV) and underwater visual census (UVC) for assessment of artificial reefs in estuaries. Journal of Experimental Marine Biology and Ecology. 2012;416–417:243–53. doi: 10.1016/j.jembe.2012.01.013

[27] Dunlop KM, Marian Scott E, Parsons D, Bailey DM. Do agonistic behaviours bias baited remote underwater video surveys of fish? Marine Ecology. 2014;36(3):810–8. doi: 10.1111/maec.12185

[28] Seitz RD, Wennhage H, Bergström U, Lipcius RN, Ysebaert T. Ecological value of coastal habitats for commercially and ecologically important species. ICES Journal of Marine Science. 2013;71(3):648–65. doi: 10.1093/icesjms/fst152

[29] Moore CG, James BD. Scoping immediate priorities for new benthic marine biological survey work in Scottish territorial waters. In: Heritage SN, editor. 2011.

[30] Ewers RM, Didham RK. Confounding factors in the detection of species responses to habitat fragmentation. Biol Rev Camb Philos Soc. 2006;81(1):117–42. doi: 10.1017/S1464793105006949 16318651

[31] Fahrig L. Effects of habitat fragmentation on biodiversity. Annual Review of Ecology, Evolution, and Systematics. 2003;34(1):487–515. doi: 10.1146/annurev.ecolsys.34.011802.132419

[32] Jackson EL, Attrill MJ, Jones MB. Habitat characteristics and spatial arrangement affecting the diversity of fish and decapod assemblages of seagrass (*Zostera marina*) beds around the coast of Jersey (English Channel). Estuarine, Coastal and Shelf Science. 2006;68(3–4):421–32. doi: 10.1016/j.ecss.2006.01.024

[33] Ricart AM, Sanmartí N, Pérez M, Romero J. Multilevel assessments reveal spatially scaled landscape patterns driving coastal fish assemblages. Mar Environ Res. 2018;140:210–20. doi: 10.1016/j.marenvres.2018.06.015 30251645

[34] Kisel Y, McInnes L, Toomey NH, Orme CDL. How diversification rates and diversity limits combine to create large-scale species-area relationships. Philos Trans R Soc Lond B Biol Sci. 2011;366(1577):2514–25. doi: 10.1098/rstb.2011.0022 21807732 PMC3138612

[35] Gilpin M, Soulé M. Minimum viable populations: processes of species extinction. Conservation Biology: the Science of Scarcity and Diversity. 1986:19–34.

[36] Collie JS, Hall SJ, Kaiser MJ, Poiner IR. A quantitative analysis of fishing impacts on shelf-sea benthos. J Anim Ecol. 2000;69(5):785–98. doi: 10.1046/j.1365-2656.2000.00434.x 29314001

[37] Collie J, Hermsen J, Valentine P, Almeida F. Effects of fishing on gravel habitats: assessment and recovery of benthic megafauna on Georges Bank. American Fisheries Society Symposium. 2005;41:325–43.

[38] Thrush SF, Dayton PK. Disturbance to marine benthic habitats by trawling and dredging: implications for marine biodiversity. Annu Rev Ecol Syst. 2002;33(1):449–73. doi: 10.1146/annurev.ecolsys.33.010802.150515

[39] Stirling DA, Boulcott P, Scott BE, Wright PJ. Using verified species distribution models to inform the conservation of a rare marine species. Diversity and Distributions. 2016;22(7):808–22. doi: 10.1111/ddi.12447

[40] Hall-Spencer J. Scallop dredging has profound, long-term impacts on maerl habitats. ICES Journal of Marine Science. 2000;57(5):1407–15. doi: 10.1006/jmsc.2000.0918

[41] Sköld M, Göransson P, Jonsson P, Bastardie F, Blomqvist M, Agrenius S, et al. Effects of chronic bottom trawling on soft-seafloor macrofauna in the Kattegat. Mar Ecol Prog Ser. 2018;586:41–55. doi: 10.3354/meps12434

[42] Grieve C, Brady D, Polet H. Review of habitat dependent impacts of mobile and static fishing gears that interact with the sea bed. Marine Stewardship Council Science Series. 2014;2:18–88.

[43] Airoldi L, Beck M. Loss, status and trends for coastal marine habitats of europe. oceanography and marine biology. Oceanography and Marine Biology - An Annual Review 2007:345-405.

[44] Halpern BS, Selkoe KA, Micheli F, Kappel CV. Evaluating and ranking the vulnerability of global marine ecosystems to anthropogenic threats. Conserv Biol. 2007;21(5):1301–15. doi: 10.1111/j.1523-1739.2007.00752.x 17883495

[45] Harris PT. Anthropogenic threats to benthic habitats. In: Harris PT, Baker E, editors. Seafloor Geomorphology as Benthic Habitat GeoHab Atlas of Seafloor Geomorphic Features and Benthic Habitats. 2nd ed. Elsevier Science; 2020. p. 35-61.

[46] Fletcher RJ Jr, Didham RK, Banks-Leite C, Barlow J, Ewers RM, Rosindell J, et al. Is habitat fragmentation good for biodiversity? Biological Conservation. 2018;226:9–15. doi: 10.1016/j.biocon.2018.07.022

[47] Haddad NM, Brudvig LA, Clobert J, Davies KF, Gonzalez A, Holt RD, et al. Habitat fragmentation and its lasting impact on Earth’s ecosystems. Sci Adv. 2015;1(2):e1500052. doi: 10.1126/sciadv.1500052 26601154 PMC4643828

[48] Hanski I. Habitat fragmentation and species richness. Journal of Biogeography. 2015;42(5):989–93. doi: 10.1111/jbi.12478

[49] South Arran Nature Conservation Marine Protected Area Order; 2014.

[50] Stewart BD, Howarth LM, Wood H, Whiteside K, Carney W, Crimmins É, et al. Marine Conservation Begins at Home: How a Local Community and Protection of a Small Bay Sent Waves of Change Around the UK and Beyond. Front Mar Sci. 2020;7. doi: 10.3389/fmars.2020.00076

[51] Natureseot. Scottish MPA Project Management Options: South Arran Possible MPA. 2014.

[52] Scottish Natural Heritage. Scottish MPA Project Management Options: South Arran Possible MPA. 2014.

[53] QGIS Development Team. QGIS Geographic Information System. 2014.

[54] SeaGIS Ltd. EventMeasure. 5.61 ed. Australia; 2019.

[55] Morris-Webb ES, Stamp TS. Biological analyses of underwater video footage from arran, loch linnhe, loch shell and loch seaforth. Journal Name or Book Title. 2015; doi: DOIorotheridentifiers

[56] O’Dell J, Bulgakova A, Amos W, Dewey S. Biological analyses of seabed imagery from within and around loch alsh, loch carron, wester ross, small isles and south arran marine protected areas in 2018. 2020;56:1–50.

[57] Coggan R, Mitchell A, White J, Golding N. Recommended operating guidelines (ROG) for underwater video and photographic imaging techniques. MESH. 2007; doi: DOIorIdentifier

[58] Hitchin R, Tuner JA, Verling E. Epibiota Remote Monitoring from Digital Imagery: Operational Guidelines. JNCC; 2015.

[59] SeaGIS Ltd. CAL. 3.30 ed. Australia; 2020.

[60] R Core Team. R: A language and environment for statistical computing. 4.2.2 ed. Vienna, Austria: R Foundation for Statistical Computing; 2022.

[61] Mitchell S, Reeve R, White T. Rdiversity. R package version 2.0. Glasgow; 2020.

[62] Hiemstra P, Pebesma E, Twenhofel C, Heuvelink G. Real-time automatic interpolation of ambient gamma dose rates from the dutch radioactivity monitoring network. Computers and Geosciences. 2008;35(8):1711–21.

[63] Bivand R, Keitt T, Rowlingson B. Rgdal: Bindings for the ‘Geospatial’ Data Abstraction Library. R package version 1.6-7. 2023.

[64] Bivand R, Rundel C. Rgeos: Interface to Geometry Engine - Open Source (‘GEOS’). 2023.

[65] Pebesma E. Multivariable geostatistics in S: the gstat package. Computers and Geosciences. 2004;30(7):683–91. doi: 10.1016/j.cageo.2004.03.012

[66] Hijmans RJ. Geosphere: Spherical Trigonometry. R package version 1.5-14 ed. 2023.

[67] Hijmans R. Raster: Geographic Data Analysis and Modeling. R package version 3.6-26 ed. 2023.

[68] Akima H, Gebhardt A, Petzoldt T, Maechler M. Akima: Interpolation of Irregularly Spaced Data. R package version 0.6-3.7 ed. 2023.

[69] Wickham H. ggplot2: Elegant Graphics for Data Analysis. Verlag, New York: Springer; 2016.

[70] Kassambara A. rstatix: Pipe-Friendly Framework for Basic Statistical Tests. R package version 0.7.2 ed. 2023.

[71] Graves S, Piepho HP, Selzer L, Dorai-Raj S. multcompView: Visualizations of Paired Comparisons. R package version 0.1-8 ed. 2019.

[72] Van der Wal J, Falconi L, Januchowski S, Shoo L, Storlie C. SDMTools: Species Distribution Modelling Tools: Tools for processing data associated with species distribution modelling exercises. R package version 1.1-221 ed. 2014.

[73] Milne BT. Measuring the fractal geometry of landscapes. Applied Mathematics and Computation. 1988;27(1):67–79. doi: 10.1016/0096-3003(88)90099-9

[74] O’Neill R, Krummel J, Gardner R, Sugihara G, Jackson B, DeAngelis D. Indices of landscape pattern. Landscape Ecology. 1988;1(3):153–62. doi: 10.1007/BF00162741

[75] St. Pierre JI, Kovalenko KE. Effect of habitat complexity attributes on species richness. Ecosphere. 2014;5(2). doi: 10.1890/ES13-00323.1

[76] Jacob C, van Bochove J, Livingstone S, White T, Pilgrim J, Bennun L. Marine biodiversity offsets: pragmatic approaches toward better conservation outcomes. Conservation Letters. 2020;13(3). doi: 10.1111/conl.12711

[77] Gill DA, Mascia MB, Ahmadia GN, Glew L, Lester SE, Barnes M, et al. Capacity shortfalls hinder the performance of marine protected areas globally. Nature. 2017;543(7647):665–9. doi: 10.1038/nature21708 28329771

[78] Edgar GJ, Stuart-Smith RD, Willis TJ, Kininmonth S, Baker SC, Banks S, et al. Global conservation outcomes depend on marine protected areas with five key features. Nature. 2014;506(7487):216–20. doi: 10.1038/nature13022 24499817

[79] Pace MC, Bailey DM, Donnan DW, Narayanaswamy BE, Smith HJ, Speirs DC, et al. Modelling seabed sediment physical properties and organic matter content in the Firth of Clyde. Earth Syst Sci Data. 2021;13(12):5847–66. doi: 10.5194/essd-13-5847-2021

[80] Moore CH, Van Niel K, Harvey ES. The effect of landscape composition and configuration on the spatial distribution of temperate demersal fish. Ecography. 2010;34(3):425–35. doi: 10.1111/j.1600-0587.2010.06436.x

[81] Bologna PAX, Heck KL. Impact of habitat edges on density and secondary production of seagrass-associated fauna. Estuaries. 2002;25(5):1033–44. doi: 10.1007/bf02691350

[82] Gorman AM, Gregory RS, Schneider DC. Eelgrass patch size and proximity to the patch edge affect predation risk of recently settled age 0 cod (Gadus). Journal of Experimental Marine Biology and Ecology. 2009;371(1):1–9. doi: 10.1016/j.jembe.2008.12.008

[83] Laurel B, Gregory R, Brown J. Settlement and distribution of Age-0 juvenile cod, Gadus morhua and G. ogac, following a large-scale habitat manipulation. Mar Ecol Prog Ser. 2003;262:241–52. doi: 10.3354/meps262241

[84] Hall-Spencer J, White N, Gillespie E, Gillham K, Foggo A. Impact of fish farms on maerl beds in strongly tidal areas. Mar Ecol Prog Ser. 2006;326:1–9. doi: 10.3354/meps326001

[85] Kamenos N, Moore P, Hall-Spencer J. Nursery-area function of maerl grounds for juvenile queen scallops aequipecten opercularis and other invertebrates. Marine Ecology Progress Series. 2004;274:183–9. doi: 10.3354/meps274183

[86] Kamenos N, Moore P, Hall-Spencer J. Small-scale distribution of juvenile gadoids in shallow inshore waters; what role does maerl play?. ICES Journal of Marine Science. 2004;61(3):422–9. doi: 10.1016/j.icesjms.2004.02.004

[87] Kaiser M, Collie J, Hall S, Jennings S, Poiner I. Modification of marine habitats by trawling activities: prognosis and solutions. Fish and Fisheries. 2002;3(2):114–36. doi: 10.1046/j.1467-2979.2002.00079.x

[88] Kamenos N, Moore P, Hall-Spencer J. Substratum heterogeneity of dredged vs un-dredged maerl grounds. Journal of the Marine Biological Association of the United Kingdom. 2003;83(3):411–3. doi: 10.1017/S0025315403007264

[89] Dorenbosch M, Grol M, Nagelkerken I, van der Velde G. Distribution of coral reef fishes along a coral reef-seagrass gradient: edge effects and habitat segregation. Mar Ecol Prog Ser. 2005;299:277–88. doi: 10.3354/meps299277

[90] Gratwicke B, Speight MR. The relationship between fish species richness, abundance and habitat complexity in a range of shallow tropical marine habitats. Journal of Fish Biology. 2005;66(3):650–67. doi: 10.1111/j.0022-1112.2005.00629.x

[91] Nagelkerken I, Dorenbosch M, Verberk W, Cocheret de la Morinière E, van der Velde G. Importance of shallow-water biotopes of a caribbean bay for juvenile coral reef fishes: patterns in biotope association, community structure and spatial distribution. Mar Ecol Prog Ser. 2000;202:175–92. doi: 10.3354/meps202175

[92] Jac C, Desroy N, Certain G, Foveau A, Labrune C, Vaz S. Detecting adverse effect on seabed integrity. Part 1: Generic sensitivity indices to measure the effect of trawling on benthic mega-epifauna. Ecological Indicators. 2020;117:106631. doi: 10.1016/j.ecolind.2020.106631

