# Supplementary figures and images for: A novel use of Stereo Baited Remote Underwater Video and Drop-Down Video for biodiversity and marine landscape mapping and prediction
Source: PLoS One. 2025 Apr 3;20(4):e0319355. doi: 10.1371/journal.pone.0319355 (PMC11967939; doi:10.1371/journal.pone.0319355)

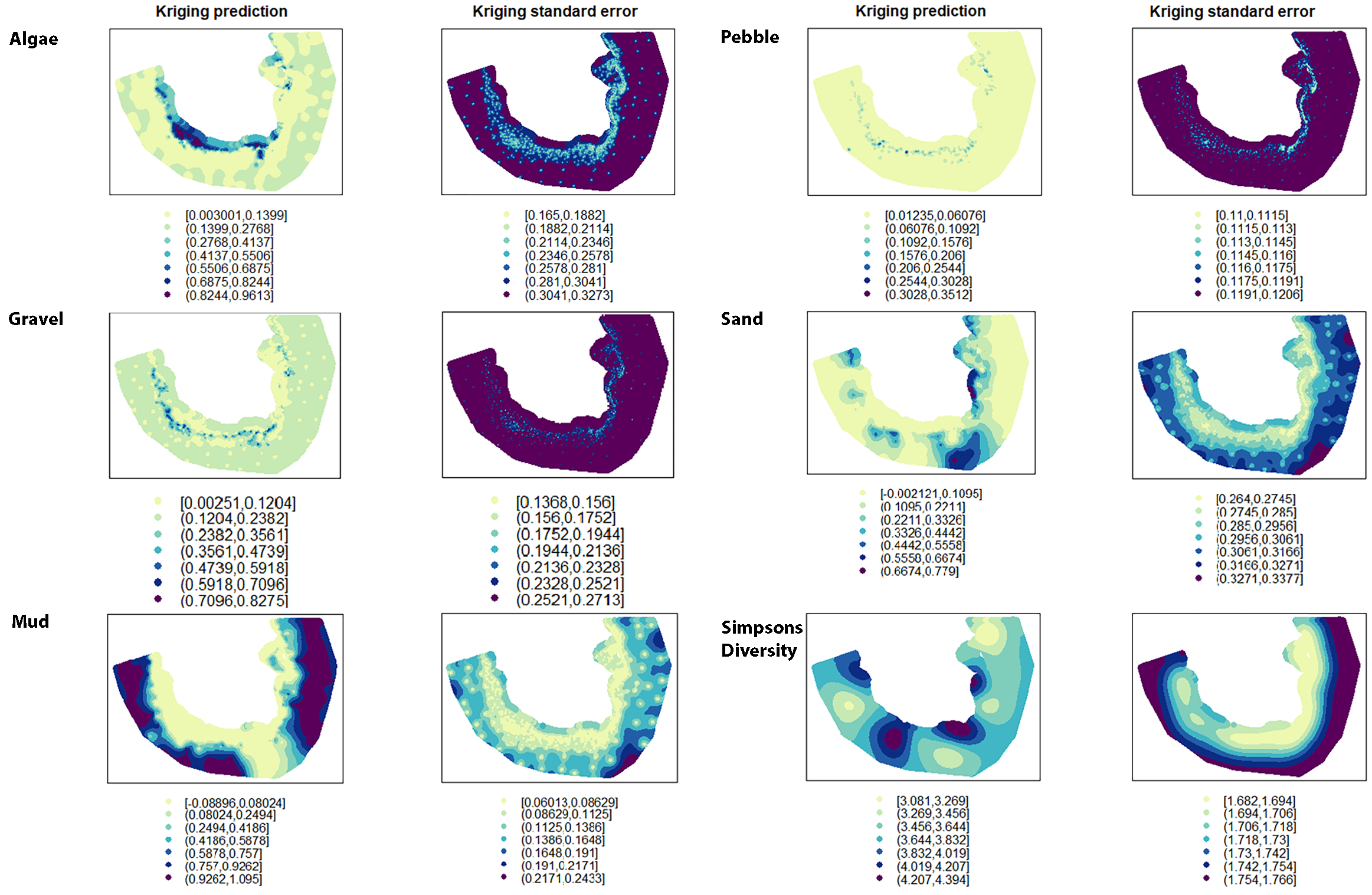

Supplement: Fig S1 — Kriged predictions of substrata coverage and corresponding kriging standard errors maps. (TIF) [file pone.0319355.s004.tif]

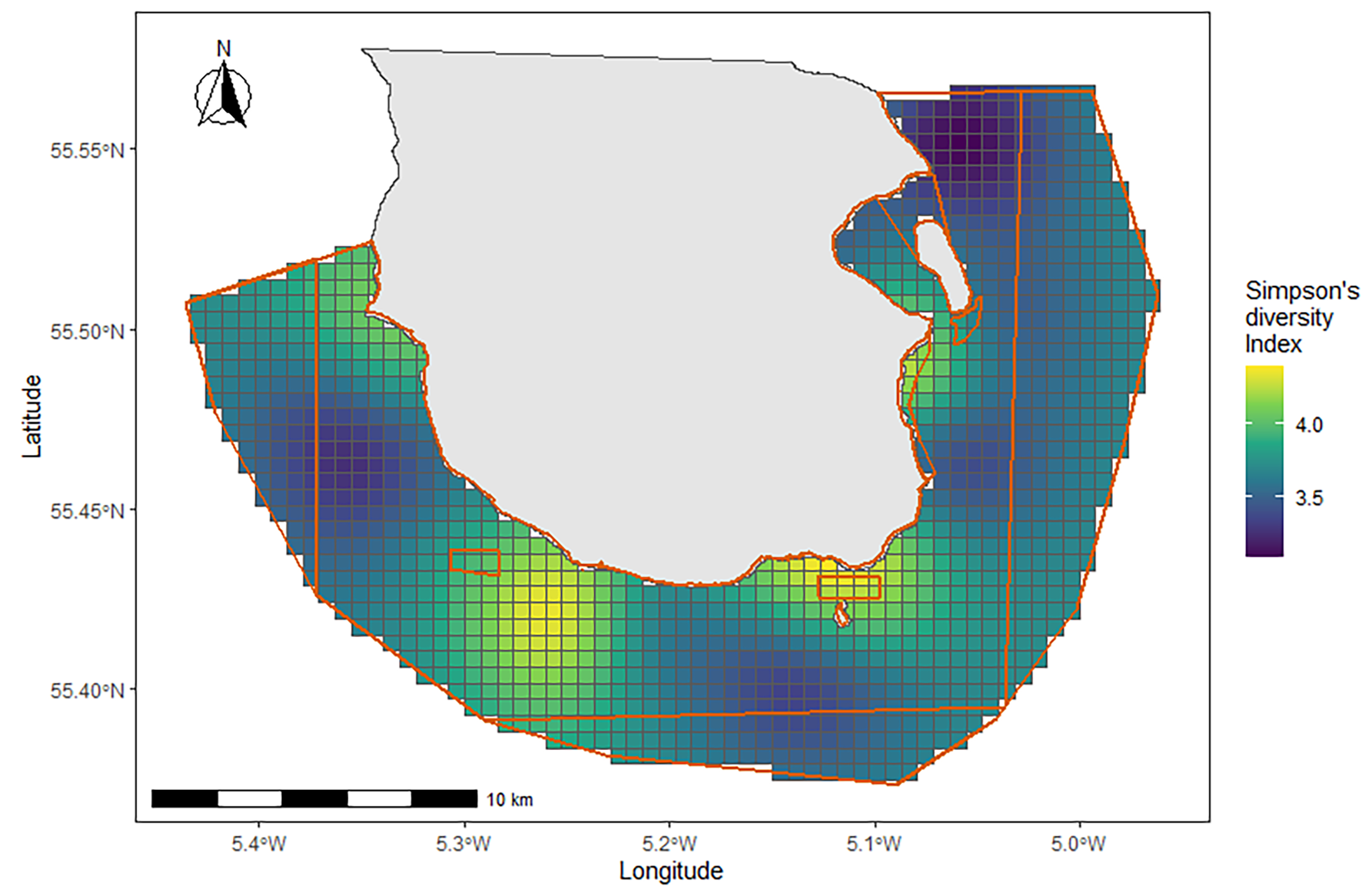

Supplement: Fig S2 — Gridded overlay of the South Arran MPA with the predicted Inverse Simpsons Diversity (Fig 7). Orange bounding boxes outline MPA fishing restrictions (as described in Table 1) (TIF) [file pone.0319355.s005.tif]
